# Drug resistance patterns and dynamics of tuberculosis in Zhejiang Province, China: Results from five periodic longitudinal surveys

**DOI:** 10.3389/fpubh.2022.1047659

**Published:** 2022-11-29

**Authors:** Lin Zhou, Beibei Wu, Fei Huang, Zhengwei Liu, Fei Wang, Mingwu Zhang, Bin Chen, Songhua Chen, Xiaomeng Wang, Yanlin Zhao

**Affiliations:** ^1^Provincial Center for TB control and prevention, Zhejiang Provincial Center for Disease Control and Prevention, Hangzhou, China; ^2^National Center for TB control and prevention, Chinese Center for Disease Control and Prevention, Beijing, China

**Keywords:** tuberculosis, China, survey, drug, resistance

## Abstract

**Background:**

As one of the high multi-drug resistance tuberculosis countries, it is critical for China to understand patterns of drug resistance to better formulate effective treatment regimens.

**Methods:**

The anti-TB Drug resistance surveillance has been conducted in Zheijang Province in years 1999, 2004, 2008, 2013, and 2018 respectively. We compared the prevalence of DR-TB from the latest survey with that of the previous four surveys in terms of all four first-line anti-TB drugs. We also examined the prevalence of rifampin-resistant TB (RR-TB) between the last two surveys and routine surveillance data.

**Results:**

Among 996 patients surveyed in 2018, the prevalence of RR-TB in new and previously treated TB cases was 2.5 and 4.3%, respectively. The prevalence of RR-TB among previously treated cases was much higher than for new cases in the four surveys from 1999 to 2013, while there was no significant difference between these groups in the 2018 survey. The percentage of TB cases resistant to fluoroquinolones in new patients was 3.8%. The prevalence of non-tuberculous mycobacteria increased over time; the prevalence of RR-TB among new cases slowly decreased. The prevalence of RR-TB in both new and previously treated TB cases from the latest two surveys was consistent with routine surveillance data.

**Conclusions:**

This consistency between routine surveillance and periodic surveys for TB cases implies that with universal testing in Zhejiang Province, data from routine surveillance could be used instead of periodic surveys to improve access to timely and appropriate treatment for DR-TB. Levels of resistance were lower than whole-country and global estimates, further indicating the value of universal drug susceptibility testing.

## Introduction

Antimicrobial resistance has become the paramount health peril for public health systems worldwide over the last two decades (1). *Mycobacterium tuberculosis*, for which drug resistance first emerged in the 1940s (2), merits being one of the highest priority pathogens, as drug-resistant tuberculosis (DR-TB) accounts for 29% of deaths attributable to antimicrobial resistance. Since the initiation in 1994 of a global project to monitor the development of DR-TB (3), 169 countries (87% of the 194 World Health Organization [WHO] Member States) have reported data on drug resistance; collectively, these countries have more than 99% of the world's population and tuberculosis (TB) cases (4). Routine testing of all patients with TB is widely recognized as the most appropriate surveillance approach for monitoring trends in drug resistance and detecting outbreaks and hotspot regions (5). More and more countries are transferring from a reliance on periodic surveys to the establishment of continuous surveillance systems based on routine drug susceptibility testing. With the expansion of rapid molecular tools, including whole genome sequencing, universal testing has the possibility of becoming a reality even in resource-limited countries (6, 7).

WHO has listed China as a high burden country for TB, TB and HIV co-infection, and multidrug-resistant TB (MDR-TB) during the period 2016–2020 (8). Yet the treatment landscape for TB has changed dramatically in recent years. In particular, the China government has strengthened and expanded implementation of rapid molecular techniques for DR-TB diagnosis in the last 5 years (9, 10). This improvement, coupled with the introduction of new anti-TB drugs, offers a critical opportunity to understand drug resistance in China and better formulate effective treatment regimens. Several provinces have recently reported their epidemiological status of DR-TB (11–13); however, they did not use the latest definition of extensively drug-resistant tuberculosis (XDR-TB) from 2021 but rather the former definition formulated by WHO in 2006 (14). Moreover, they did not compare the results of periodic surveys with routine surveillance.

To address the above issues, our study analyzed drug-resistance patterns within the TB epidemic in Zhejiang Province—the first province in China to launch sentinel surveillance and conduct drug resistance surveys following initiation of the WHO/International Union Against Tuberculosis and Lung Disease global anti-TB drug resistance surveillance project in 1994. Zhejiang Province was also the first province in China to conduct periodic surveys for DR-TB since WHO integrated the country into its surveillance network for DR-TB in 1999.

To our best knowledge, our study is the first to address the different prevalence patterns of RR-TB among new and previously treated TB cases over the past two decades in a well-developed province with a cascade of drug resistance surveys. With the implementation and improvement of DR-TB control and prevention in Zhejiang Province, the routine surveillance should be able to reflect the real epidemic of DR-TB. Our findings, using the latest definition of DR-TB from WHO, could provide insights for the province to estimate anti-TB drug procurement as well as improve interventions for TB control and prevention.

## Methods

### Study design and participants

We undertook a longitudinal analysis of data from the 1999, 2004, 2008, 2013, and 2018 anti-TB drug resistance surveys in Zhejiang Province, which is in the southeast of China with a total population of 57 million. In accordance with WHO protocol, each of the five surveys was a cross-sectional survey conducted with the same method in the same 30 counties randomly sampled from the 90 counties in Zhejiang Province, which could reflect the appropriate and representative population (13, 15).

For the 2018 survey, the sample size of new TB cases was 30, according to probability-proportional-to-size sampling. Patients who were newly diagnosed as sputum smear-positive since January 1, 2018, were eligible for inclusion and patients with the history of DR-TB were excluded. All eligible patients were enrolled sequentially until 30 new patients were enrolled in each site. All the enrollment was completed before the end of 2018. The survey obtained ethics approval from Zhejiang Provincial Center for Disease Control and Prevention (CDC) and written informed consent from patients.

### Procedures

Trained physicians at the sites gave all eligible TB patients involved in this study a questionnaire on their treatment history. Patients under 14 years of age or with intellectual disabilities were interviewed together with their guardians. The questionnaire was then double-checked by another trained physician. In the event of inconsistences, the local county CDC reconfirmed the questionnaire responses by interviewing patients again, visiting TB patients' family members, and inquiring of their medical record. All questionnaire confirmed by local county CDC will be submitted to the provincial CDC every week, and the provincial CDC will randomly selected the questionnaires for rechecking again before entering into an electronic database in parallel.

Each presumptive TB case were asked to provide three sputum samples for sputum smear microscopy, and the morning and evening samples were collected in their home and the spot samples was collected in the hospital. Two samples were cultured by solid Löwenstein-Jensen media in each site as well. Drug susceptibility testing was performed at provincial TB reference laboratory using the proportion method, and results were compared with results for standard strain. The quality of provincial reference laboratories is ensured and evaluated annually by the national reference laboratory of the China CDC.

### Statistical analysis

Cochran-Armitage trend test was used for categorical data, and the risk factors associated with DR-TB were examined by a multivariate logistic regression model. All statistical tests were two-tailed, and the *p*-value < 0.05 was considered significant. All tests were done by Base R (version 3.6.3).

## Results

### Patients

A total of 996 cases were enrolled in the survey in 2018; of these, 78 cultures (7.8%) were not recovered successfully. Of the 918 cases with a positive mycobacterial culture, 81 (8.8%) were nontuberculous mycobacteria (NTM), the left 837 (91.2%) acquired drug susceptibility testing (DST) results. Among these with DST, 768 were new cases, and 69 were previously treated cases (Table 1). The percentage of new cases increased significantly, from 84.6% in 1999 to 91.8% in 2018 (*p* < 0.0001), and the percentage of NTM increased significantly as well, from 2.6% in 1999 to 8.8% in 2018 (*p* < 0.0001).

**Table 1:** TB case distribution by five surveys in Zhejiang Province, 1999–2008.

**Year**	**Total sample** **size**	***Mycobacterium tuberculosis***			**NTM** **(%)**	**Others** **(%)**
		**New cases (%)**	**Previously treated cases (%)**	**Subtotal (%)**		
1999	1,013	807 (84.6)	147 (15.4)	954 (97.4)	25 (2.6)	34 (3.4)
2004	1,066	846 (84.3)	158 (15.7)	1,004 (97.1)	30 (2.9)	32 (3.0)
2008	1,077	842 (89.8)	96 (10.2)	938 (96.4)	35 (3.6)	104 (9.7)
2013	1,010	842 (90.0)	94 (10.0)	936 (94.8)	51 (5.2)	23 (2.3)
2018	996	768 (91.8)	69 (8.2)	837 (91.2)	81 (8.8)	78 (7.8)

NTM, non-tuberculous mycobacteria; Others, specimens were not recovered successfully.

### Drug-resistant tuberculosis in 2018

In the latest survey, 74.0, 80.9, and 97.5% of new TB cases were susceptible to all nine anti-TB drugs, all four first-line drugs, and rifampin, respectively, while 66.7, 75.4, and 95.7% of previously treated TB cases were susceptible to all nine anti-TB drugs, all four first-line drugs, and rifampin, respectively. Overall, 2.5% of new cases and 4.3% of previously treated cases were rifampin-resistant TB (RR-TB), of which 81.8% were multidrug-resistant TB (MDR-TB—i.e., resistant to both rifampin and isoniazid) (Table 2).

**Table 2:** Drug resistance patterns of eight anti-TB drugs in Zhejiang Province, 2018.

**Resistance patterns**	**New TB cases** **(*****N*** = **768)**		**Previously treated TB cases (*****N*** = **69)**	
	**No**.	**% (95 CI)**	**No**.	**% (95 CI)**
Susceptible to nine anti-TB drugs	568	74.0 (70.9–77.1)	46	66.7 (55.5–77.8)
Susceptible to four first-line drugs	621	80.9 (78.1–83.6)	52	75.4 (65.2–85.5)
Susceptible to rifampin	749	97.5 (96.4–98.6)	66	95.7 (90.8–100)
Susceptible to isoniazid	699	91.0 (89.0–93.0)	61	88.4 (80.9–96.0)
Resistant to isoniazid (Hr-TB)	50	6.5 (4.8–8.3)	5	7.2 (1.1–13.4)
Resistant to fluoroquinolones	29	3.8 (2.4–5.1)	3	4.3 (0.0–9.2)
Resistant to rifampin (RR-TB)	19	2.5 (1.4–3.6)	3	4.3 (0-9.2)
Susceptible to isoniazid	3	0.4 (0–0.8)	1	1.4 (0–4.3)
Resistant to isoniazid (MDR-TB)	16	2.1 (1.1–3.1)	2	2.9 (0–6.9)
Susceptible to all other drugs	3	0.4 (0–0.8)	1	1.4 (0–4.3)
Resistant to fluoroquinolones (pre-XDR)	6	0.8 (0.2–1.4)	0	-
Resistant to cycloserine	4	0.5 (0–1)	0	-
Resistant to ethambutol	13	1.7 (0.8–2.6)	1	1.4 (0–4.3)
Resistant to aminoglycosides	3	0.4 (0–0.8)	0	-
Resistant to prothionamide	2	0.3 (0–0.6)	0	-
Resistant to p-aminosalicylic acid	4	0.5 (0–1)	0	-

CI, confidence interval.

Among the new cases with susceptibility to rifampin, 3.8% were resistant to fluoroquinolones, higher than the 2.5% of new cases with resistance to rifampin (*p* < 0.0001). Among the patients with MDR-TB, 18.8% (3/16) of new cases and 50.0% (1/2) of previously treated cases were susceptible to all the other five anti-TB drugs, 37.5% (6/16) of new cases had additional resistance to fluoroquinolones (so-called pre-XDR), and 81.3% (13/16) of new cases were resistant to ethambutol.

### Factors linked to drug-resistant tuberculosis

Multivariate analysis showed that all factors but living with TB cases from the family had no association with RR-TB. Patients with TB who lived with any TB cases from the family had about a four-fold higher chance of acquiring RR-TB than those who did not live with TB cases from the family (Table 3).

**Table 3:** Characteristics of rifampin-resistant TB by multivariate analysis in Zhejiang Province, 2018.

**Factors**	**Estimate**	**Standard error**	***p*-value**	**Odds ratio (95% CI)**
Intercept	2.7594	0.6515	< 0.0001	
Gender				
Male	ref	ref	ref	ref
Female	0.1243	0.4973	0.8026	1.1 (0.4–3.0)
Age group				
< 25 years	ref	ref	ref	ref
25–44 years	−0.7658	0.3534	0.0502	0.4 (0.1–1.8)
45–64 years	0.4071	0.4596	0.3063	1.4 (0.2–8.2)
≥65 years	0.1294	0.4620	0.7793	1.0 (0.2–5.8)
Address				
Local	ref	ref	ref	ref
Migrant	−0.2137	0.5066	0.6732	0.8 (0.3–2.2)
Diabetes				
No	ref	ref	ref	ref
Yes	−0.4358	0.6708	0.4220	0.6 (0.2–2.4)
Treatment history				
No	ref	ref	ref	ref
Yes	−0.7631	0.5848	0.1919	0.5 (0.1–1.5)
Household contacts				
No	ref	ref	ref	ref
Yes	1.3681	0.6002	0.0226	3.9 (1.2–12.7)
Hepatitis B				
No	ref	ref	ref	ref
Yes	−1.0849	0.8191	0.1854	0.3 (0.1–1.7)

ref, reference category in dummy coding.

### Epidemic trends of drug-resistant tuberculosis

**Figure 1:**
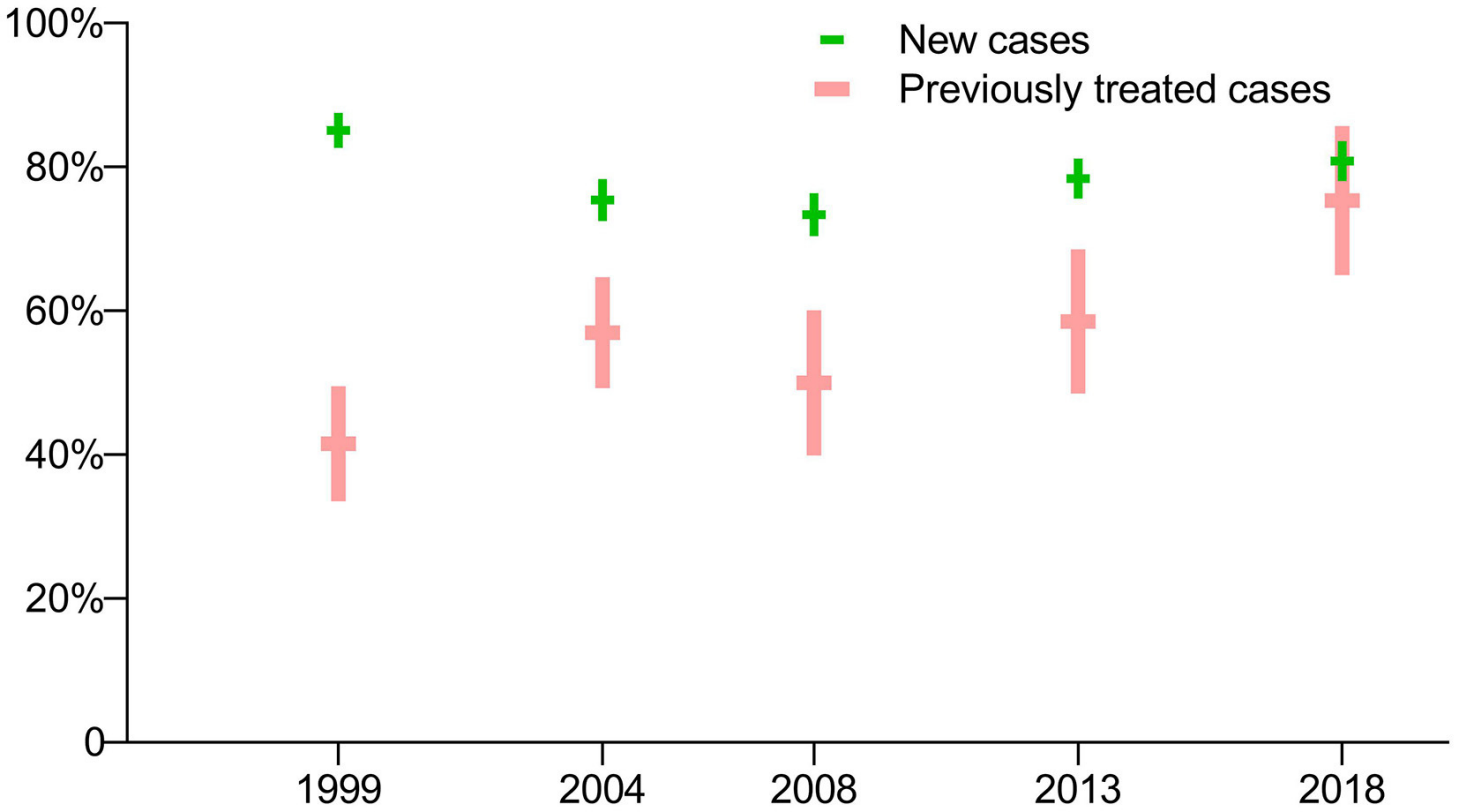
Prevalence of TB cases susceptible to all four first-line anti-TB drugs: breakdown by treatment history in five periodic surveys.

The prevalence of susceptibility to all four first-line anti-TB drugs was much higher for new TB cases than for previously treated cases in the first four drug-resistance surveys (*p* < 0.0001), while there was no significant difference between the two groups in the latest survey in 2018 (*p* = 0.2705). The prevalence of both new and previously treated cases susceptible to all four first-line anti-TB drugs has steadily increased since the third survey, in 2008 (Figure 1).

**Figure 2:**
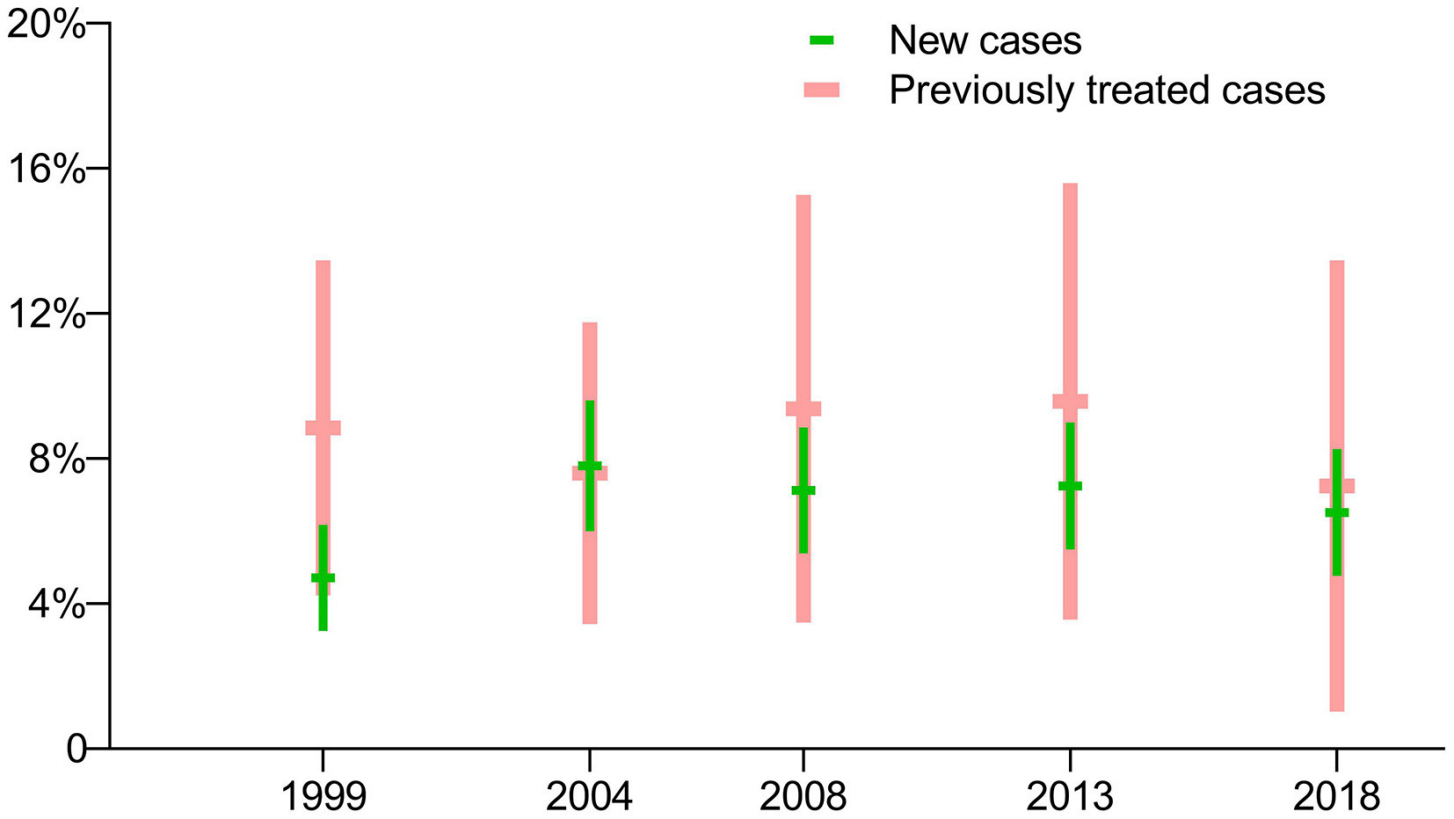
Prevalence of isoniazid-resistant TB: breakdown by treatment history in five periodic surveys.

The prevalence of isoniazid-resistant TB (Hr-TB) with new cases of TB was lower than for previously treated cases in the first survey in 1999 (*p* = 0.0404), but there were no significant differences in the latest four surveys (*p* > 0.05). The prevalence of Hr-TB with new cases of TB had no significant difference among the five surveys (*p* = 0.1140), as did the prevalence of Hr-TB with previously treated cases (*p* = 0.9762) (Figure 2).

**Figure 3:**
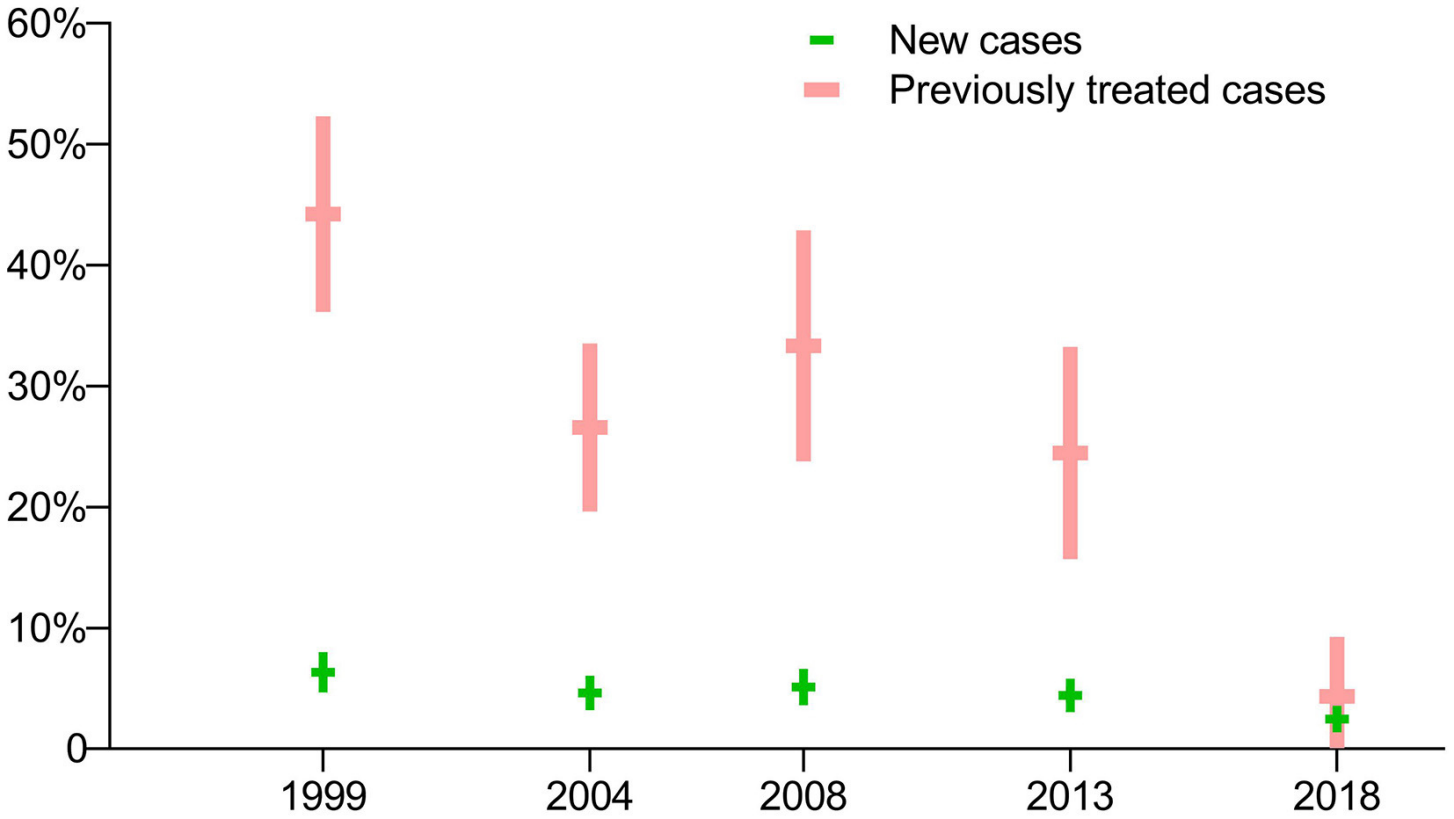
Prevalence of rifampin-resistant TB: breakdown by treatment history in five periodic surveys.

The prevalence of RR-TB with previously treated cases was much higher than for new cases in the first four surveys (*p*<*0*.0001), while there was no significant difference between these groups in the latest survey in 2018 (*p* = 0.2705). In general, the prevalence of RR-TB among both new and previously treated cases showed a downward trend (Figure 3).

**Figure 4:**
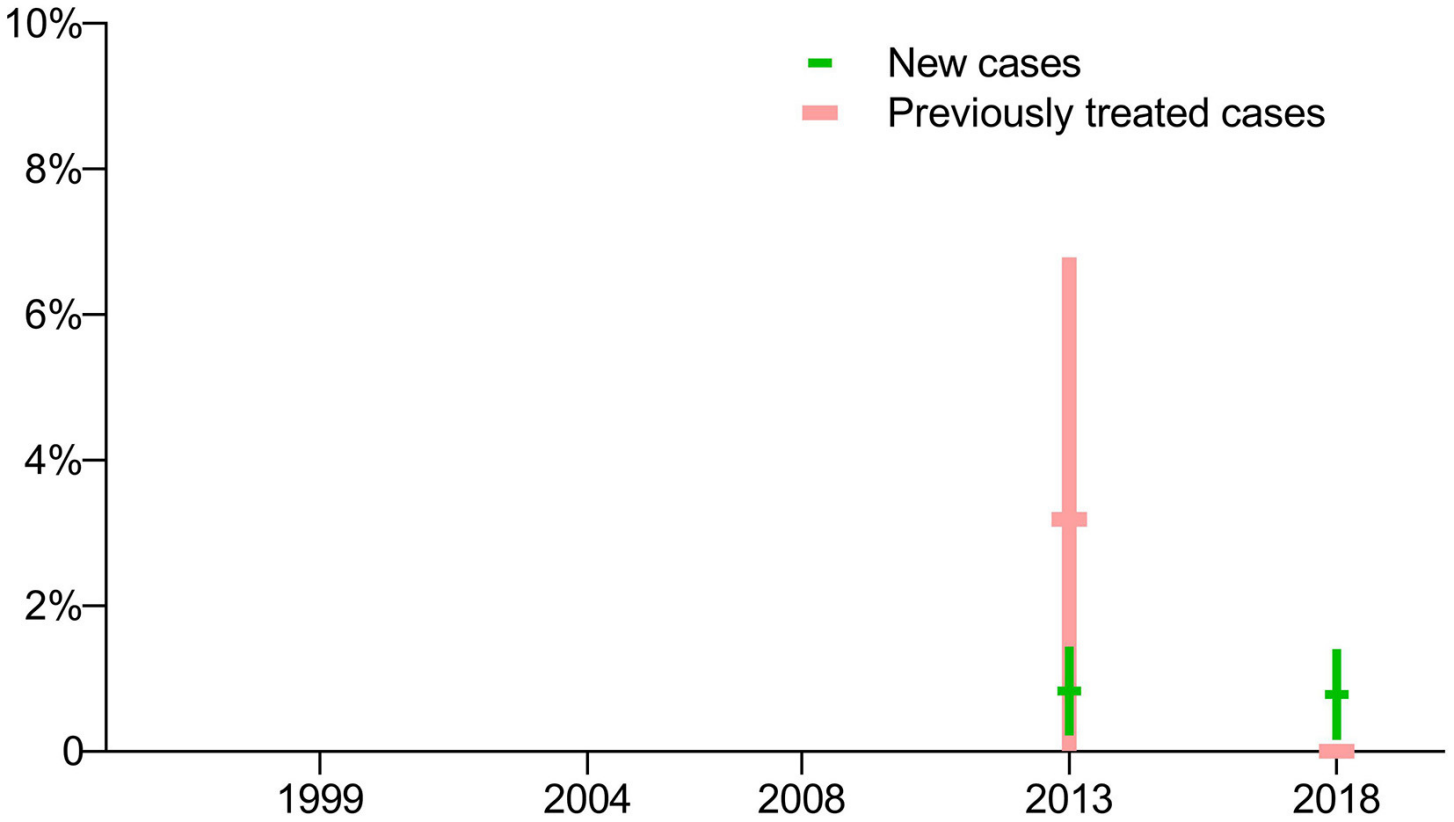
Prevalence of pre-extensively drug-resistant TB: breakdown by treatment history in five periodic surveys.

Drug susceptibility testing was not conducted for fluoroquinolones in the first three surveys, thus data on the prevalence of fluoroquinolone-resistant TB are not available for those years. In terms of the latest two surveys, there was no significant difference in the prevalence of pre-XDR-TB among new cases, but there was a sharp decrease in the prevalence of pre-XDR-TB among previously treated cases (Figure 4).

### Comparison with routine surveillance data

**Figure 5:**
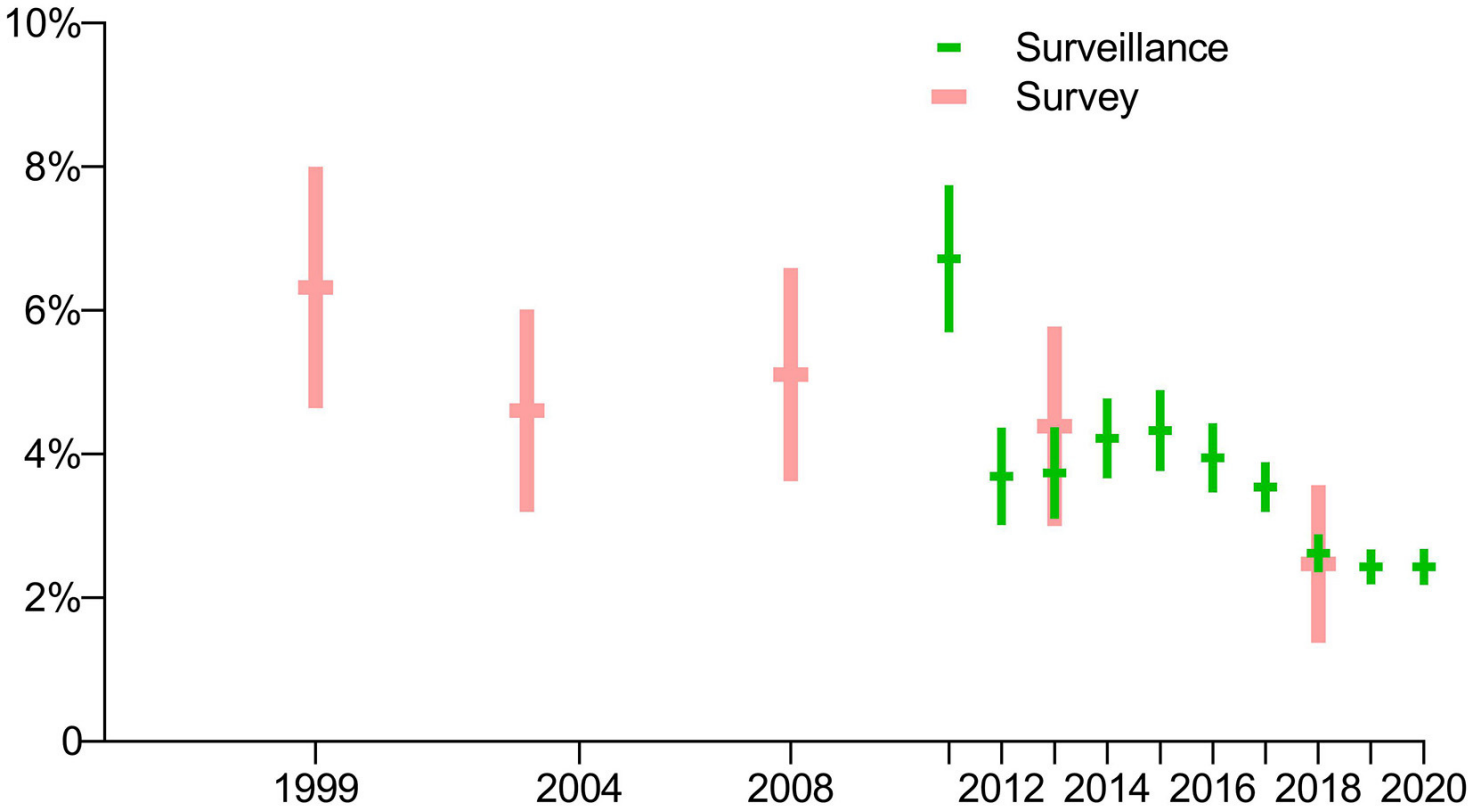
Comparison of prevalence of rifampin-resistant TB among new cases in periodic surveys and routine surveillance.

The prevalence of rifampin resistance among new cases in 2013 and 2018 from the routine surveillance system was 3.7% (95% CI, 3.1–4.4) and 2.6% (95% CI, 2.4–2.9), respectively, and had no significant difference compared with the surveys conducted those years (*p* = 0.3786 and 0.7992, respectively). According to routine surveillance data, after a drop in 2012, the prevalence of RR-TB increased slightly through 2013 before peaking in 2015, decreasing through 2018, and reaching its lowest point in 2019 (Figure 5).

**Figure 6:**
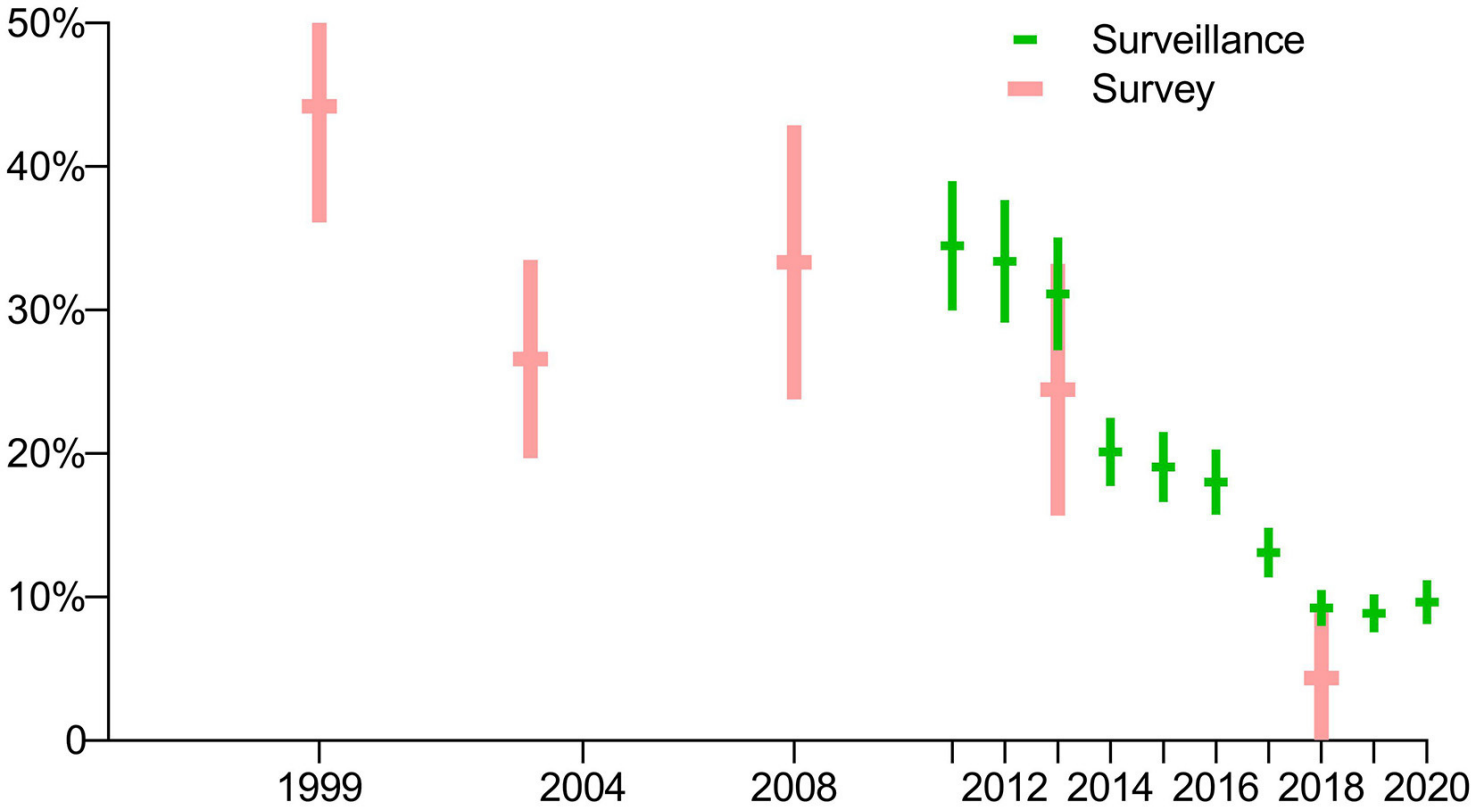
Comparison of prevalence of rifampin-resistant TB among previously treated cases in periodic surveys and routine surveillance.

The prevalence of rifampin resistance among previously treated cases in 2013 and 2018 as per the routine surveillance system was 31.1% (95% CI, 27.2–35.1) and 9.2% (95% CI, 8.0–10.5), respectively, and had significant difference between them (*p* = 0.0005). The prevalence of rifampin resistance among previously treated cases in the survey of 2013 and 2018 was 24.5% (95% CI, 15.8–33.2) and 4.3% (95% CI, 0.0–9.2), respectively, and had no significant difference compared with the routine surveillance system those years (*p* = 0.1944 and 0.1646, respectively). In general, the prevalence of RR-TB has declined since 2011 (Figure 6).

## Discussion

Drug resistance in new cases normally implies a transmission control problem, whereas in previously treated cases it reflects on the treatment process, either poor compliance or irrational regimens. Accordingly, the slowly decreasing prevalence of RR-TB among new cases in Zhejiang Province suggests that primary resistance is being driven by ongoing transmission with few changes, while the sharp drop in prevalence of RR-TB among previously treated cases indicates high treatment adherence and good treatment outcomes over the past 5 years. The findings reflect the importance of infection control and treatment adherence, which should be delivered by both the physicians to TB patients and CDCs to social mass.

RR-TB is the most important indicator of MDR-TB and has implications for treatment regimens. The latest drug-resistance survey in Zhejiang Province presents a much lower prevalence of RR-TB (2.5% in new cases and 4.3% in previously treated cases) than the average prevalence in China (7.1% in new cases and 21.0% in previously treated cases) as well as globally (3.4% in new cases and 18.0% in previously treated cases) in 2018 (16).

Several possible reasons could contribute to this. First, non-compliance with the chemotherapy regimen and misuse or misadministration of anti-TB drugs often leads to emergence of drug-resistant strains (17), but with the transition of the China TB service system, Zhejiang Province has been able to guarantee affordable, qualified TB clinical care for accurate diagnosis, rational treatment regimen, and adequate treatment course since 2011 (18). This has led to the sharp decline in the prevalence of acquired DR-TB. Second, with support from the Global Fund to Fight AIDS, Tuberculosis and Malaria during 2008–2012, followed by China central government funding since 2013, Zhejiang Province scaled up drug-susceptibility testing across the whole province. The directive for all DR-TB suspects to get drug-susceptibility testing led to the detection of most RR-/MDR-TB cases and a reduction in transmission.

According to several previous studies, the estimated proportion of MDR-TB resulting from transmission accounts for the majority of cases and varies substantially with different countries' notification data, ranging from 48 to 99% (19–22). The national survey of DR-TB in China in 2008 indicated that primary transmission accounted for 80% of MDR-TB cases (23). Another study in China further suggested that recent transmission of MDR-TB strains helped drive the MDR-TB epidemic in Shanghai City, which is adjacent to Zhejiang Province (24). The current universal drug-susceptibility testing coverage for all TB patients and the low prevalence of RR-TB in Zhejiang highlights the importance of shortening the diagnosis delay and strengthening hospitalization of RR-TB cases until sputum conversion to negative. Preventive treatment of MDR-TB close contacts with TB infections is also currently recommended (25). Both of these measures can decrease transmission.

The prevalence of susceptibility to all four first-line anti-TB drugs among both new and previously treated TB cases increased since the third survey, which was consistent with the declining prevalence of RR-TB. Although only the last two surveys had drug-susceptibility testing results for fluoroquinolones, the prevalence of pre-XDR-TB among new and previously treated TB cases followed the same pattern as that of RR-TB with the same reasons mentioned above. However, the higher percentage resistant to fluoroquinolones in new TB cases than resistant to rifampin was likely due to the wide use of fluoroquinolones for antibiotic therapy in China. Hr-TB is the most common form of DR-TB worldwide (26); however, the prevalence of Hr-TB in Zhejiang Province varied from 4.7 to 7.8% in new cases and from 7.2 to 9.6% in previously treated cases, which remained stable, with no significant difference in prevalence between new and previously treated TB cases in the latest four surveys, suggesting that previous TB combination therapy is unlikely to contribute to the prevalence. With the rollout of molecular tests, Hr-TB is likely to be more commonly diagnosed in the coming years (27).

The latest survey observed that living with TB cases from the family was the only risk factor linked to DR-TB (odds ratio = 3.9, 95% CI 1.2–12.7). This was different from the first four surveys, where previous treatment was the strongest predictor for MDR-TB (odds ratio = 10.9, 95% CI 9.4–12.7) (13). As the first four surveys showed a higher prevalence of RR-TB in previously treated TB cases than that in new TB cases, previous TB treatment was not surprisingly a predictor for MDR-TB, a trend reflected in previous studies (28). Accordingly, stopping the transmission of RR-TB strains should be prioritized in the future.

Our analysis also indicates that NTM prevalence is increasing over time, which is valuable information for clinicians using molecular identification methods for suspicious MDR-TB. Other studies have also suggested an increase in the prevalence rates of NTM over the last four decades (29). Previous studies indicated that Bacille Calmette-Guérin (BCG) vaccination confers cross-protection against NTM, thus in countries without a nationwide BCG immunization program, a rise in NTM cases is expected (30–33). However, BCG is compulsory for all newborns in China since 1978, and BCG coverage in Zhejiang Province in 2017 was more than 90% (34). This clearly indicates that the increased trend of NTM is happening despite BCG coverage. Additional studies have shown that the incidence and prevalence of pulmonary NTM is associated with a range of underlying health conditions, such as immunosuppression, age, sex, previous history of lung disease, and increased incidence of chronic lung disease (35, 36). This correlation needs to be further studied as it relates to the increasing trend of NTM in Zhejiang Province.

This study had a number of limitations. First, the survey was not designed to estimate the resistance to new anti-TB drugs; namely, bedaquiline and linezolid. Therefore, the study could not present a full picture of the drug-resistance epidemic in Zhejiang Province. Second, the study was designed as a cluster sample survey with a probability-proportional-to-size sampling, meaning that not all TB patients in the sites in 2018 were recruited; however, the survey was designed to be representative of the entire TB patient population in Zhejiang Province. Third, the method used for the drug-resistance surveys was conventional phenotypic testing rather than a molecular-based method, which has inherent limitations and is less reliable for second-line drugs.

Continuous antimicrobial resistance surveillance is an essential part of national containment strategies (37). The last result of our study was the remarkable consistency between routine surveillance data and periodic drug-resistance surveys for both new and previously treated TB cases, which suggests that Zhejiang Province, a setting with universal access to drug susceptibility testing, could use data from routine surveillance instead of periodic surveys to improve access to timely and appropriate treatment and care for DR-TB. Finally, as the shorter, all-oral, bedaquiline-containing MDR-TB regimen is likely to increase in the coming years (38, 39), drug susceptibility testing for fluoroquinolones should be added to the routine surveillance network in Zhejiang Province while continuing to monitor for rifampicin resistance. In the future, molecular technologies, including high-throughput sequencing-based technologies, should be scaled up to replace conventional phenotypic testing in drug resistance surveillance (40).

## Data availability statement

The raw data supporting the conclusions of this article will be made available by the authors, without undue reservation.

## Ethics statement

The studies involving human participants were reviewed and approved by Ethics Committees of Zhejiang Provincial Center for Disease Control and Prevention. Written informed consent to participate in this study was provided by the participants' legal guardian/next of kin.

## Author contributions

SC, XW, and YZ conceived of the presented idea. ZL, FW, MZ, and BC carried out the study and collected data. FH wrote the manuscript with support from LZ and BW. All authors contributed to the article and approved the submitted version.

## Funding

This study was supported by China CDC-Tuberculosis Control and Prevention Project (228711).

## Conflict of interest

The authors declare that the research was conducted in the absence of any commercial or financial relationships that could be construed as a potential conflict of interest.

## Publisher's note

All claims expressed in this article are solely those of the authors and do not necessarily represent those of their affiliated organizations, or those of the publisher, the editors and the reviewers. Any product that may be evaluated in this article, or claim that may be made by its manufacturer, is not guaranteed or endorsed by the publisher.
